# Self-care and healthcare seeking practices among patients with hypertension and diabetes in rural Uganda

**DOI:** 10.1371/journal.pgph.0001777

**Published:** 2023-12-11

**Authors:** Andrew K. Tusubira, Isaac Ssinabulya, Robert Kalyesubula, Christine K. Nalwadda, Ann R. Akiteng, Christine Ngaruiya, Tracy L. Rabin, Anne Katahoire, Mari Armstrong-Hough, Evelyn Hsieh, Nicola L. Hawley, Jeremy I. Schwartz

**Affiliations:** 1 Uganda Initiative for Integrated Management of Non-Communicable Diseases, Kampala, Uganda; 2 Department of Community Health and Behavioural Sciences, School of Public Health, College of Health Sciences, Makerere University, Kampala, Uganda; 3 Uganda Heart Institute, Mulago Hospital Complex, Kampala, Uganda; 4 Department of Internal Medicine, College of Health Sciences, Makerere University, Kampala, Uganda; 5 Departments of Physiology and Internal Medicine, College of Health Sciences, Makerere University, Kampala, Uganda; 6 African Community Center for Social Sustainability (ACCESS), Nakaseke, Uganda; 7 Yale Network for Global Non-Communicable Diseases, Yale University, New Haven, Connecticut, United States of America; 8 Department of Emergency Medicine, Stanford School of Medicine, Palo Alto, California, United States of America; 9 Department of Internal Medicine, Yale School of Medicine, New Haven, Connecticut, United States of America; 10 Child Health and Development Centre, Makerere University, Kampala, Uganda; 11 Department of Social & Behavioral Sciences, School of Global Public Health, New York University, New York, United States of America; 12 Department of Epidemiology, School of Global Public Health, New York University, New York, United States of America; 13 Department of Chronic Disease Epidemiology, Yale School of Public Health, New Haven, Connecticut, United States of America; Universiti Malaya, MALAYSIA

## Abstract

**Background:**

Implementing effective self-care practices for non-communicable diseases (NCD) prevents complications and morbidity. However, scanty evidence exists among patients in rural sub-Saharan Africa (SSA). We sought to describe and compare existing self-care practices among patients with hypertension (HTN) and diabetes (DM) in rural Uganda.

**Methods:**

Between April and August 2019, we executed a cross-sectional investigation involving 385 adult patients diagnosed with HTN and/or DM. These participants were systematically randomly selected from three outpatient NCD clinics in the Nakaseke district. Data collection was facilitated using a structured survey that inquired about participants’ healthcare-seeking patterns, access to self-care services, education on self-care, medication compliance, and overall health-related quality of life. We utilized Chi-square tests and logistic regression analyses to discern disparities in self-care practices, education, and healthcare-seeking actions based on the patient’s conditions.

**Results:**

Of the 385 participants, 39.2% had only DM, 36.9% had only HTN, and 23.9% had both conditions (HTN/DM). Participants with DM or both conditions reported more clinic visits in the past year than those with only HTN (P = 0.005). Similarly, most DM-only and HTN/DM participants monitored their weight monthly, unlike those with only HTN (P<0.0001). Participants with DM or HTN/DM were more frequently educated about their health condition(s), dietary habits, and weight management than those with only HTN. Specifically, education about their conditions yielded adjusted odds ratios (aOR) of 5.57 for DM-only and 4.12 for HTN/DM. Similarly, for diet, aORs were 2.77 (DM-only) and 4.21 (HTN/DM), and for weight management, aORs were 3.62 (DM-only) and 4.02 (HTN/DM). Medication adherence was notably higher in DM-only participants (aOR = 2.19). Challenges in self-care were significantly more reported by women (aOR = 2.07) and those above 65 years (aOR = 5.91), regardless of their specific condition(s).

**Conclusion:**

Compared to rural Ugandans with HTN-only, participants with DM had greater utilization of healthcare services, exposure to self-care education, and adherence to medicine and self-monitoring behaviors. These findings should inform ongoing efforts to improve and integrate NCD service delivery in rural SSA.

## Introduction

The growing burden of non-communicable diseases (NCDs), particularly hypertension (HTN) and diabetes mellitus (DM), in sub-Saharan Africa (SSA), threatens already overburdened health systems [1]. SSA health systems, primarily oriented toward acute and episodic care, remain ill-equipped to support ongoing care for chronic conditions [2, 3]. In such settings where resources are limited and access to critical care is a challenge, prevention of chronic disease progression and mitigation of complications benefits from active participation of the patient through self-care [4, 5].

Self-care is the ability of an individual, in conjunction with family, community, and healthcare professionals to manage symptoms, treatments, lifestyle changes, psychosocial, cultural, and spiritual consequences of health conditions [6]. The process of self-care includes understanding one’s condition, monitoring one’s perceived health status, implementing strategies to promote adherence to lifestyle and medicines, and identifying and managing symptoms [7]. Promoting self-care for NCD control in low-resource settings benefits both patients and health systems by shifting fundamental care tasks from providers to patients, empowering patients, and enhancing well-being [8]. Little is, however, known about how those living with NCDs in SSA engage in self-care, particularly in rural settings where health system challenges associated with staffing and medicine supply chains are amplified.

In Uganda, the prevalence of both HTN and DM are increasing and most patients with these conditions experience suboptimal glycemic and/or blood pressure control [9–11]. Efforts to improve the care and associated health outcomes of these conditions that share risk factors and frequently co-occur are ongoing, albeit inconsistently implemented. These include integrated care clinics, patient support groups, education to promote self-care, and equipping community health workers to support patients with NCDs [12–14]. Improving the reach and fidelity of self-care could be a cost-effective, and proactive approach to the prevention and treatment of these increasingly common conditions [15].

While self-care offers numerous advantages, studies from SSA indicate that people with NCDs are not sufficiently engaged in it [16]. Recently conducted studies in rural Uganda highlighted how patients with HTN or DM adopted diverse self-care practices based on specific symptoms [17–19]. This paper delves into the self-care behaviors and healthcare-seeking tendencies of patients in rural Uganda diagnosed with HTN and/or DM. A comprehensive analysis of such practices for prevalent NCDs is essential to devise strategies that promote self-care and enhance patient outcomes. Recognizing that disease severity and self-care requirements can vary based on individual or combined conditions, we’ve also scrutinized the differences in self-care practices among patients with HTN, DM, or both HTN and DM.

## Materials and methods

### Study design and site

We conducted a cross-sectional study at three health centers in Nakaseke District from April to August 2019. Nakaseke is located in central Uganda, approximately 66 km north of Kampala, the capital city, and has an estimated population of 191,100 residents [20]. Nakaseke is home to the African Community Center for Social Sustainability (ACCESS), a community-based organization that serves as a Center of Excellence for NCDs in rural Uganda [21]. The organization, among other activities, coordinates NCD clinics at three local health facilities (Nakaseke General Hospital, Semuto Health Centre IV, and Nakaseke LifeCare Center) on alternating days of the week. The three health facilities provide healthcare services to a catchment area beyond Nakaseke district to include “greater Luwero region” which encompasses several counties of surrounding districts like Luwero, Nakasongola and Wakiso. These NCD clinics offer an integrated approach for both DM and HTN whereby patients attend the same clinic and receive care, for one or both conditions, from a clinical group trained in diagnosis and management. At the time of this study, weekly patient volume at these clinics was approximately 75 at Nakaseke General Hospital, 38 at Semuto Health Centre and 25 at Nakaseke LifeCare Center. These clinics constituted the recruitment sites for the current study.

### Study population and sample

We recruited adult patients (above 17 years) with HTN, DM, or HTN/DM attending outpatient NCD clinics at these three health facilities. Eligible participants should have been diagnosed at least three months before enrollment to ensure that they were able to reflect on their experiences with facility-based care and self-care practices. We, however, exclude those who were critically ill and were unable to provide written informed consent.

Our sample size was 385 patients based on power calculations using the Leslie Kish formula (n = (z^2^pq)/d^2^)) [22], with parameters: 1.96 standard normal deviation(z); 5% precision(d) and 50% estimated proportion(p) for patients following recommended self-care behaviors/practices. Since the actual proportion of patients in this population with DM and/or HTN engaging in self-care is unknown, we used 50%, based on maximum variation [23]. Probability-proportional-to-size sampling was used to determine the number of participants selected from each health facility. Together with the lead clinicians at the NCD-clinics, we used patient registers to determine the number of patients enrolled and expected to be attending these facilities—481 at the time of data collection. The sample size for each facility was proportionally allocated based on patient volume. This resulted in a target sample size of 272 patients from Nakaseke General Hospital, 60 patients from Semuto Health Centre, and 53 patients from Nakaseke LifeCare Clinic.

At each facility, participants were selected using systematic random sampling. The approximate number of patients with HTN or DM expected on each clinic day was used to calculate the sampling interval. The sampling interval therefore differed each clinic day in each facility and across facilities. Patients were sampled and recruited while in the waiting area and were invited to participate in the study. All patients who were invited during sampling agreed to participate in the study.

### Data collection

Seven trained research assistants with a nursing background, and were unaffiliated with the health facilities, collected the data under supervision of the first author. We translated the structured questionnaire into Luganda, the most spoken local language in Nakaseke district, and pre-tested it among patients with HTN, DM, and HTN/DM attending outpatient care at a rural, public sector health center outside of Nakaseke. We then made appropriate revisions to enhance clarity and streamline administration. Research assistants administered face-to-face interviews in private rooms, at the health facilities, in either English or Luganda.

### Measures

In addition to the socio-demographic characteristics (e.g., age, gender), our tool included the following domains: 1) Self-care practice outcomes including medication practices, alcohol consumption, smoking, and physical activity; 2) Healthcare seeking behavior; 3) exposure to self-care education/information; and 4) health-related quality of life (HRQoL).

### Self-care practice outcomes

While self-care is a broad concept that encompasses what patients get engaged in to manage the condition, treatments, including lifestyle changes [6], self-care practices in this study comprised four dependent outcomes practices for self-management and care: adherence to medications, level of physical activity, smoking and alcohol use. These practices are conventionally recommended (overlapping) for both HTN and DM self-care. Questions about adherence to medications, smoking cessation, and alcohol consumption, were taken from the Summary of Diabetes Self-care Assessment Scale (DSCA)” for DM [24] and from the “Hypertension Self-care Activity Level Effects Scale (H-scale)” for HTN [25, 26]. These tools provide similar measurement for medication adherence, nonsmoking, and alcohol consumption. We captured data self-reported data on medicine adherence, which included questions about how often a participant took their medicines in the past seven days, including recommended dosage and timing. Participants who had taken their medicines on all the past seven days, as prescribed, were categorized as adherent to medication. Participants who reported 0-day smoking in the past seven days were categorized as nonsmoking and a three-item scales assessed alcohol consumption and those who did not consumer are considered abstainers. Level of physical activity was assessed using the International Physical Activity Questionnaire Short Form (IPAQ-SF) [27].

### Healthcare seeking behaviors

These are characterized by the proactive measures that patients take to access services and resources essential for self-care. Crucial aspects within this category encompass accessibility to self-care services or equipment and the introduction to self-care education. To gauge this access, we gathered data on the self-reported number of clinic visits in the past year. Questions regarding self-care services included the availability of a weighing scale and a blood pressure machine outside the clinic. Furthermore, we evaluated how often participants checked their weight and blood pressure.

### Exposure to self-care education/information and medication practices

This domain included questions about receipt of disease-specific self-care related education, healthy diet, and weight management.

### Health-related quality of life (HRQOL) assessment

We used the EQ-5D-5L tool to measure HRQOL, a validated instrument frequently applied in low-resource settings [28]. This tool encompasses five specific dimensions: 1) mobility, 2) self-care, 3) usual activities, 4) pain/discomfort, and 5) anxiety/depression. Each dimension has five levels of severity: no problems, slight problems, moderate problems, severe problems, and extreme problems [28].

### Statistical analysis

Descriptive analyses were conducted to obtain frequencies and percentages for the socio-demographic characteristics and the variables under the self-care related domains. Comparative analyses between HTN-only, DM-only, and HTN/DM, were conducted using Chi-square test to assess differences among demographic characteristics, healthcare seeking, and access to services for self-care. The analysis of variance ANOVA (with adjustment for multiple comparisons using Tukey’s P-value) was run to determine differences in age (normally distributed continuous variable) across the three conditions. Logistic regression was used to model the association between five outcomes: a) receipt of education on condition; b) education on a healthy diet; c) education on weight management; d) adherence to medications; e) self-care related problems and the three condition as the predictors of interest while adjusting for age, sex, and level of education as other key covariates selected basing on how they performed in univariate analysis. Multicollinearity checks were conducted as well as likelihood ratio tests to identify the best fitting models. Analyses were conducted using Stata Statistical Software: (Release 14), College Station, TX: StataCorp LP.

### Human subjects and ethical approval

We received ethical approval from the Makerere University School of Medicine Ethics Review Committee (REF 2019–060), the Yale Human Subjects Committee (2000025066), and the Uganda National Council of Science and Technology (HS2698). Administrative clearance was also obtained from the Nakaseke District Health Office and from the three health facilities. All participants provided written informed consent prior to participation in study activities.

## Results

### Demographic characteristics of the participants

The mean age of participants was 54.0 years (SD: ±14.6). Most participants, 67% (257/385), were women; 60% (230/385) resided in Nakaseke district; 59% (227/385) were married; and 52% (201/385) reported farming as their occupation. Although 80% (310/385) had some level of formal education, more than half, 56% (215/385) had attained only primary level education. The largest number of participants (39.2%; 151/385) had DM-only; 36.9% (142/385) had HTN-only; and 23.9% (92) had HTN/DM.

HTN-only, DM-only, and HTN/DM varied significantly by age (*p<0*.*0001*); sex (*p = 0*.*005*); district of residence (*p<0*.*0001*); marital status (*p = 0*.*012*); and level of education (*p = 0*.*003*). Participants with DM-only were significantly younger (mean age:48.0±14.9 years) than participants with HTN-only (mean age:58.33±14.6 years; *p<0*.*0001*) and HTN/DM (mean age:57.3±9.8 years; *p<0*.*0001*). Although the number of women was higher than for men in all conditions, women comprised more than three quarters (78%; 72/92) of participants with HTN/DM compared to 68% (97/142) women with HTN-only and 58% (88/151) DM-only women. While 75% (106/142) of HTN-only participants and 60% (55/92) HTN/DM were residents of Nakaseke district, participants with DM-only were evenly split between Nakaseke (46%; 69/151) and neighboring Luwero district (44%; 67 /151). More than 80% of the participants with DM-only (83%; 125/151) and HTN/DM (87%; 80/92) had attained at least primary level education compared to 74% (105/142) of patients with HTN-only (p = 0.003) *(Table 1)*.

**Table 1:** Demographic characteristics of the participants.

*Variable*	HTN-only N = 142	DM-only N = 151	HTN/DM N = 92	*p-value*
**Age** *n (%)*				
Mean (Standard deviation)	58.3 (±14.6)	48.0 (±14.9)	57.3 (±9.8)	^A^ <0.0001 *
**Age categories** *n (%)*				<0.0001 *
18–29	3 (2.1)	22 (14.6)	0 (0.0)	
30–45	25 (17.6)	41 (27.1)	10 (10.9)	
46–55	29 (20.4)	40 (26.5)	36 (39.1)	
56–65	37 (26.1)	30 (19.9)	32 (34.8)	
>65	48 (33.8)	18 (11.9)	14 (15.2)	
**Sex** *n (%)*				0.005 *
Men	45 (31.7)	63 (41.7)	20 (21.7)	
women	97 (68.3)	88 (58.3)	72 (78.3)	
**District of residence** *n (%)*				< 0.0001 *
Luwero	30 (21.1)	67 (44.4)	32 (34.8)	
Nakaseke	106 (74.6)	69 (45.7)	55 (59.8)	
Other districts	6 (4.2)	15 (9.9)	5 (5.4)	
**Marital status** *n (%)*				0.012 *
Single	8 (5.6)	17 (11.3)	2 (2.2)	
Married (includes cohabiting)	81 (57.0)	91 (60.3)	55 (59.8)	
Divorced/separated	18 (12.7)	26 (17.2)	17 (18.5)	
Widowed	35 (24.6)	17 (11.3)	18 (19.6)	
**Education level** *n (%)*				0.003 *
No education	37 (26.1)	26 (17.2)	12 (13.0)	
Primary	69 (48.6)	83 (55.0)	63 (68.5)	
Secondary	26 (18.3)	39 (25.8)	16 (17.4)	
Tertiary	10 (7.0)	3 (2.0)	1 (1.1)	
**Occupation** *n (%)*				0.861
Farmer	73 (51.4)	78 (51.7)	50 (54.3)	
Formally employed	25 (17.6)	33 (21.9)	13 (14.1)	
Casual laborer	5 (3.5)	7 (4.6)	3 (3.3)	
Unemployed	39 (27.5)	33 (21.8)	26 (28.3)	
**Monthly Household income**^**@**^ *n (%)* **(USD)**				0.289
Less than approx.13USD	24 (16.9)	33 (21.9)	20 (21.7)	
Approx. 13-65USD	30 (21.1)	27 (17.9)	16 (17.4)	
Approx. 66–134 USD	8 (5.6)	19 (12.6)	8 (8.7)	
Approx. 135 USD and above	10 (7.0)	10 (6.6)	2 (2.2)	
Not sure/ don’t know	70 (49.3)	62 (41.1)	46 (50.0)	

* P-value < 0.05; ^A^ = One-way ANOVA test statistic (F P-value)

**Acronyms:** HTN = hypertension; DM = diabetes mellitus; USD = United States Dollar

^**@**^ Units were converted from Ugandan Shillings (UGX) to USD (using 3700UGX:1USD exchange rate) for presentation of data, though we collected data using local currency.

### Healthcare seeking and access to services for self-care

Differences by condition existed among the number of clinic visits over the last twelve months, and frequency of weight and blood pressure monitoring over the last month. More than 70% (109/151) of DM-only and 80% (74/92) HTN/DM participants had attended the clinic seven to 14 times over the preceding twelve months compared to 61% (86/142) of HTN-only participants (P = 0.005). Also, the majority of DM-only (97%, 146/151) and HTN/DM (98%, 90/92) participants checked their weight at least once over the prior month compared to 78%, 111/142) of HTN-only participants (P*< 0*.*0001*). No differences existed in access to equipment for self-care (Table 2).

**Table 2:** Healthcare seeking and access to services and equipment for self-care.

Variable	HTN-only *(n = 142) (%)*	DM-only *(n = 151) (%)*	HTN/DM *(n = 92) (%)*	*p-value*
**Number of clinic visits over 12 months**				*0*.*005* *
1 to 6	56 (39.4)	44 (29.1)	18 (19.6)	
7 to 14	86 (60.6)	107 (70.9)	74 (80.4)	
**Physical Activity levels**				0.196
Low	34 (23.9)	27 (17.8)	25 (27.2)	
Moderate	30 (21.1)	25 (16.6)	20 (21.7)	
High	78 (54.9)	99 (65.6)	47 (51.1)	
**Current smoker**				0.979
Yes	3 (2.1)	2 (1.3)	1 (1.1)	
No	139 (97.9)	149 (98.7)	91 (98.9)	
**Alcohol use in last 7 days**				0.594
Yes	16 (11.3)	12 (8.0)	10 (10.9)	
No	126 (88.7)	139 (92.0)	82 (89.1)	
**Frequency of checking weight in past month**				*< 0*.*0001* *
More than once over the month	17 (12.0)	17 (11.3)	4 (4.4)	
Once over the month	94 (66.2)	129 (85.4)	86 (93.5)	
Not checked in the past month	31 (21.8)	5 (3.3)	2 (2.1)	
**Frequency of checking BP over the last month**				*< 0*.*0001* *
More than once over the month	32 (22.5)	NA	5 (5.4)	
Once over the month	110 (77.5)	NA	87 (94.6)	
**Access to weighing scale outside the clinic**				*0*.*093*
Yes, at home	3 (2.1)	11 (7.3)	5 (5.4)	
Yes, at another place	6 (4.2)	14 (9.3)	5 (5.4)	
No	133 (93.7)	126 (83.4)	82 (89.2)	
**Access to BP machine outside the clinic**				*0*.*160*
Yes, at home	6 (4.2)	NA	1 (1.1)	
Yes, at another place	20 (14.1)	NA	8 (8.7)	
No	116 (81.7)	NA	83 (90.2)	

* P-value < 0.05 (significant). **Acronyms:** HTN = hypertension; DM = diabetes mellitus; NA = not applicable; BP = blood pressure.

### Differences in self-care-related education and adherence to medications

Significant differences existed between the three disease groups in multiple domains related to health education and adherence to medications. Compared to those with HTN-only, participants with DM were more likely to receive education about their specific condition (DM-only: aOR = 5.57 CI:2.72, 11.41; HTN/DM: aOR = 4.12; CI:1.90, 8.93); healthy diet (DM-only: aOR = 2.77; CI: 1.34, 5.71; HTN/DM: aOR = 4.21; CI: 1.74, 10.66); or weight management (DM-only: aOR = 3.62; CI: 1.73, 7.49; HTN/DM: aOR = 4.02; CI: 1.76, 9.18). Lastly, DM-only participants were twice as likely to report adherence to medications compared to participants with HTN-only (aOR = 2.19; CI: 1.16, 4.11). There was no significant difference in adherence to medicines when comparing HTN/DM and HTN-only (Table 3).

**Table 3:** Unadjusted and adjusted estimates for self-care education domains and adherence to medications.

*Variable*	Self-care domains			
	**Been educated about your condition**			
	**Yes** *(n = 312) (%)*	***No*** *(n = 73) (%)*	***Crude OR (95% CI)***	***Adjusted OR (95% CI)***
HTN-only	93 (29.8)	49 (67.1)	1.0	1.0
DM-only	137 (43.9)	14 (19.2)	5.16 (2.69, 9.87) *	5.57 (2.72, 11.41) *
HTN/DM	82 (26.3)	10 (13.7)	4.32 (2.06, 9.07) *	4.12 (1.90, 8.93) *
	*Adjusted for*: *age*, *sex*, *level of education*			
	**Educated about a healthy diet**			
	**Yes** ***(n = 331) (%)***	***No*** ***(n = 54) (%)***		
HTN-only	110 (33.2)	32 (59.3)	1.0	1.0
DM-only	136 (41.1)	15 (27.8)	2.64 (1.36, 5.12) *	2.77 (1.34, 5.71) *
HTN/DM	85 (25.7)	7 (12.9)	3.53 (1.48, 8.39) *	4.21 (1.74, 10.66) *
	*Adjusted for*: *age*, *sex*, *level of education*			
	**Educated about weight management**			
	**Yes** ***(n = 321) (%)***	***No*** ***(n = 64) (%)***		
HTN-only	100 (31.2)	42 (65.6)	1.0	1.0
DM-only	138 (42.9)	13 (20.3)	4.46 (2.27, 8.74) *	3.62 (1.73, 7.49) *
HTN/DM	83 (25.9)	9 (14.1)	3.87 (1.78, 8.42) *	4.02 (1.76, 9.18) *
	*Adjusted for*: *age*, *sex*, *level of education*			
	**Adherence to medications**			
	**Yes** ***(n = 297) (%)***	***No*** ***(n = 88) (%)***		
**Condition**				
HTN-only	105 (33.4)	37 (42.1)	1.0	1.0
DM-only	128 (43.1)	23 (26.1)	1.96 (1.20, 3.51) *	2.19 (1.16, 4.11) *
HTN/DM	64 (21.5)	28 (31.8)	0.81 (0.45, 1.44)	0.95 (0.51, 1.76)
**Sex**				
Men	108 (36.4)	20 (22.7)	1.0	1.0
Women	189 (63.6)	68 (77.3)	0.51 (0.30, 0.90) *	0.63 (0.34, 1.14)
			*Adjusted for*: *age*, *level of education*	

* P-value < 0.05 (significant)

### Health-related quality of life

Considering the five EQ-5D-5L dimensions of HRQoL, significant statistical differences were only present for the dimension of self-care (*P = 0*.*004*). In the unadjusted estimates, DM-only participants were less likely to have problems with self-care compared to HTN-only participants (crude OR = 0.45; CI:0.26, 0.77) and no difference when compared to those with HTN/DM (cO.R = 1.11; CI:0.64, 1.92). However, in the adjusted model, participants aged 66 years and above were five times more likely to have problems with self-care compared to those between 18 and 29 years (aOR = 5.91; CI:1.24,28.06). Although not significant in the model, the odds of having self-care problems increased with increase in age from aOR = 1.58; CI:0.32, 7.75 for those aged 30–45; aOR = 2.63; CI:0.56, 12.40 for those aged 46–55; aOR = 3.77; CI:0.80, 17.68 for those aged 56–65. Women participants were twice as likely to report having problems with self-care than men (aOR = 2.07; CI:1.21, 3.56) (Table 4).

**Table 4:** Unadjusted and adjusted estimates for self-care related problems among patients with hypertension (HTN) and diabetes (DM) in rural Uganda.

Variable	Reported self-care problems N = 112 (%)	No self-care problem N = 273 (%)	Crude OR (95% CI)	Adjusted OR (95% CI)
**Condition**				
HTN-only	49 (43.8)	93 (34.1)	1.0	1.0
DM-only	29 (25.9)	122 (44.7)	0.45 (0.26, 0.77) *	0.65 (0.37, 1.15)
HTN/DM	34 (30.3)	58 (21.2)	1.11 (0.64, 1.92)	1.13 (0.63, 2.02)
**Age categories**				
18–29	2 (1.8)	23 (8.4)	1.0	1.0
30–45	13 (11.6)	63 (23.1)	2.37 (0.49, 11.33)	1.58 (0.32, 7.75)
46–55	29 (25.9)	76 (27.8)	4.39 (0.97, 19.80)	2.63 (0.56, 12.40)
56–65	32 (28.6)	67 (24.5)	5.49 (1.22, 24.74) *	3.77 (0.80, 17.68)
Above 65	36 (32.1)	44 (16.1)	9.41 (2.08, 42.62) *	5.91 (1.24, 28.06) *
**Sex**				
Men	25 (22.3)	103 (37.7)	1.0	1.0
Women	87 (77.7)	170 (62.3)	2.11 (1.27, 3.50) *	2.07 (1.21, 3.56) *

* P-value < 0.05 (significant)

## Discussion

In this study, we sought to describe self-care and healthcare seeking practices among patients living with HTN and/or DM in rural Uganda and to explore differences in self-care by condition. This study adds to a growing literature regarding HTN and DM health service delivery and self-care in Uganda and SSA [17, 18, 29]. Across multiple domains, significant statistical differences existed between participants based on their condition. In this study, compared to HTN-only, participants with DM-only and those with HTN/DM had more frequent contact with healthcare providers, more frequent weight monitoring, greater exposure to condition-specific health education, dietary and weight management education, and greater self-reported medication adherence. Irrespective of condition, older participants and women were more likely to report problems with self-care.

Even when these NCD-clinics give monthly appointments to all patients, there were more DM patients attending integrated clinics compared to HTN patients in this study, which is not expected given the estimated national prevalence of HTN is far greater than that of DM [9, 30]. A possible reason could be the asymptomatic nature of HTN, often called the ‘silent killer’ [31]. The less intrusive symptoms among HTN-only patients might lead to reluctance or lower their motivation to overcome barriers to adherence to clinic appointments. A systematic review that investigated causes of non-adherence to HTN treatment in 16 countries showed that some patients with HTN think that absence of symptoms indicates normalization of blood pressure and, hence, less need for continued treatment, decreasing healthcare seeking [32]. Moreover, despite the access barrier that transport typically represents in such settings, more DM-only participants than HTN, in this study, traveled from outside Nakaseke District to seek NCD care. This finding might suggest that DM-only patients perceive a benefit from traveling for care including perceived higher quality of care or medication availability [12] and is supported by literature on healthcare seeking among rural dwellers [33, 34]. Further research should be conducted to better understand these differences in healthcare seeking.

We found greater reported medicine adherence among participants with DM-only or HTN/DM, compared to those with HTN only. This finding could perhaps, further, indicate differences in how the severity of DM is perceived and patients’ response to symptoms faced. Patients who believe that they are at risk of severe disease outcomes tend to have greater symptom awareness and self-care practices [35]. A study in rural Uganda reported that patients with DM perceived DM as a severe disease due to its numerous health effects and incurability [36]. Another Ugandan study showed how patients with DM expressed deep worry and lost hope due to the condition however, appreciated the potential for improved quality of life with consistent treatment [37]. Given the greater medicine adherence gap in patients with HTN-only, effective innovative strategies should be introduced to motivate medicine adherence across persons with NCDs irrespective of the disease or symptom severity experienced.

Perhaps because of more frequent clinic attendance, participants with DM had greater exposure to condition-specific health education than participants with HTN-only. This greater exposure likely contributes to the higher self-reported medicine adherence among the DM subgroups. Our recent findings from another study in Nakaseke district found that patients with HTN, more than those with DM or both conditions, desired clinics with health education; this corresponds with the finding in our current study that participants with HTN only were less likely to have had exposure to disease-specific education [29]. While both HTN and DM patients in this community receive health education at these facilities and through community outreach focusing on both diseases [38], strategies to reduce discrepancies in exposure to health education between HTN and DM patients should be implemented. Further exploration can be done to understand the reasons why patients with HTN are less likely to be offered education at their clinic visits in this rural setting. An understanding of one’s chronic health condition represents a major upstream component of self-care, without which the awareness of symptoms and the motivation to self-manage and seek care are limited [39]. These differences between disease subgroups in terms of exposure to self-care education and healthcare seeking choices represent an opportunity for improvement and future work.

Our findings reveal that reported problems with self-care were higher among older patients (above 64 years) irrespective of their disease. Studies have shown that older adults with chronic NCDs have unique self-care challenges that can impair daily activities, increase risk of depression, and worsen perceived symptoms, all of which can negatively impact HRQoL [40, 41]. Mechanisms to improve self-care in older patients with NCDs might require availing opportunities to increase their knowledge about their condition and offer tailored self-care support [42]. In this study, self-care problems were also higher among women patients. Unique barriers including financial constraints to overcome the healthcare costs, gender inequality, and women’s family responsibilities have been documented to challenge women’s access to NCD care in developing countries [43]. Our recent parallel qualitative study among patients with HTN and/or DM in Nakaseke revealed the importance of instrumental and emotional support for self-care from family and peer networks [17]. All these findings might suggest an interaction between age and gender with social networks and connectedness. Further research is needed to further elucidate the factors that might create obstacles for self-care and negatively impact HRQoL among older women patients in rural settings. This work should be regarded within the context of some limitations. The participants were recruited from health facilities, which means these patients may face fewer barriers to accessing care and were more likely to have invested in self-care. Therefore, the results may not be generalizable to the total population of persons with HTN and/or DM including those who are not accessing or not enrolled in clinical care. Also, some stand-alone conventional self-care practices, (including foot care, eye care, reduced sugar foods/drink for DM and reduced sodium diet for HTN) were not measured.

## Conclusion

In our study, multiple self-care domains and health seeking behaviors varied by chronic condition. Notable differences were in the domains of adherence to medicines, frequency of contact with healthcare providers, self-monitoring practices, and health education. Greater problems with self-care were seen among older women participants. The presence of DM appeared to drive greater or improved adherence to appropriate self-care and more frequent utilization of healthcare services. It is therefore important to further understand and leverage the factors that are driving better DM self-care and health seeking behavior as Uganda and other SSA countries aim to improve and integrate NCD care. To optimize integrated services, programs should place attention on increasing uptake of self-care across NCDs and learning from successful strategies already being used by patients. In health systems in which integrated care delivery is increasingly being sought, these findings may offer a metric of success and highlight opportunities for continued improvement.

## Supporting information

S1 Table De-identified data set. (XLSX)
